# Women at medical conferences 2016 – still hitting their head at the glass ceiling

**DOI:** 10.1007/s00508-016-1157-8

**Published:** 2017-01-13

**Authors:** Karin Amrein, Simone Stoisser, Magdalena Hoffmann

**Affiliations:** Austria, Department of Internal Medicine, Division of Endocrinology and Diabetology, Medical University of Graz, Auenbruggerplatz 15, 8036 Graz, Austria

From Austria to Australia, women continue to be a rare species as faculty of medical conferences although in many countries, medical students and young doctors are more often women than men [1, 2]. This is a problem for women in science, because such meetings can be extremely productive, many successful projects have once started in restricted access, such as faculty lounges or at faculty dinners during such events. The underrepresentation of women in academic medicine is particularly evident in some subspecialties such as critical care and emergency medicine [3]. For example, the annual European Society for Emergency Medicine (EuSEM) congress was recently held in Vienna. According to data presented there, the percentage of women among speakers and moderators was only 20.9%, although the total female input including delegates and poster presenters was almost twice as high: 39.3%. According to one of the organizers, simply not enough high-profile women could be found. This is a simple but devastating statement. Is it true? Of course not, certainly not in an international context. In the last years, hundreds of scientists/speakers have decided to boycott “all-men panels” in different fields and offered their help in finding female speakers (e. g. [4]). This is an interesting approach because more often than not, the development of the scientific program of a conference is a relatively informal process that favors “old boys clubs”. Sometimes academics directly suggest giving a talk at a future congress, a possibility of which (according to our personal experience) many women seem to be unaware of. Such unwritten laws and non-transparent processes have been shown to disadvantage women. The most striking example that women need to work harder for similar academic recognition is *Nepotism and Sexism in Peer Review* showing that a female researcher needed 2.5 times as many publications as a male researcher to achieve the same score at the Swedish Medical Research Council [5].

Social media in critical care and emergency medicine (SMACC) is a revolutionary idea including the concept of Free Open Access Medical Education (FOAM). Annual meetings started a few years ago and it would now likely be the largest medical conference in the world if tickets were not strictly limited. The last conference took place in Dublin in June 2016 and the organizers have shown the world how it is possible to steeply increase the percentage of women faculty to almost 40% even in these traditionally male dominated specialties: look hard and encourage promising and enthusiastic but likely younger than usual women to give a talk at such a prestigious meeting. Undoubtedly, the organizing committee will be able to find many suitable women again next June in Berlin at DAS SMACC [6]. FeminEM, an open access resource aiming to support the development and advancement of all women in emergency medicine, has recently opened a speaker’s bureau for exactly this purpose [7]. Certainly, the social media challenge the traditional stereotypes and perceptions in science faster than ever before (#ilooklikeascientist, #ilooklikeaprofessor).

Why is this important? Besides the simple reason of fairness, it would be illogical to recruit only from half of the possible candidates. Also, it is a powerful (discouraging) message to young aspiring women scientists if they see few or no women at the top of their fields although many of their peers are women suggesting that the pipeline is still quite leaky. A disproportionate number of qualified women drop out of science in the early stages and the desire to leave seems to be directly related to the impact of a science career on family life [8, 9]. Unfortunately, the biologically and scientifically (re)productive years coincide unfavourably, and many women at the stage of their career when they are potential speakers at congresses have children. Then it becomes increasingly challenging to organize a trip to a conference. If the children stay at home, it is often difficult or impossible when no grandparents are near; if the children come along, it is expensive and it may overall even be worse as only very few conferences currently offer on-site child care. The same is rarely true for male scientists who are also fathers. Today, it is still mostly women who take over multiple roles (and this is also why women who work part-time still often have less free time and >100 h-weeks). Then there is also a phenomenon called “motherhood penalty” – it seems that the fact alone of being a mother is perceived as a disqualification [10].

We are unable to offer quick solutions, but with this report, we want to raise the awareness for this systematic problem and invite congress committees to publish their female representation at different levels. Being invited to medical conferences comes with many opportunities, and it is crucial for younger women to see female role models when they consider staying in academia. We want to encourage conference organizers to think about creative solutions to identify potential female faculty, and women to “lean in” and to actively seek out and accept challenges. More women are now present in academia than ever before – there are enough excellent women who are already or have the potential to be great faculty, and they are not all that difficult to find.
